# Confocal Characterization of Intestinal Dendritic Cells from Myxines to Teleosts

**DOI:** 10.3390/biology11071045

**Published:** 2022-07-12

**Authors:** Alessio Alesci, Gioele Capillo, Angelo Fumia, Emmanuele Messina, Marco Albano, Marialuisa Aragona, Patrizia Lo Cascio, Nunziacarla Spanò, Simona Pergolizzi, Eugenia Rita Lauriano

**Affiliations:** 1 Department of Chemical, Biological, Pharmaceutical and Environmental Sciences, University of Messina, 98166 Messina, Italy; emmanuele.messina@outlook.com (E.M.); malbano@unime.it (M.A.); plocascio@unime.it (P.L.C.); spergolizzi@unime.it (S.P.); elauriano@unime.it (E.R.L.); 2 Department of Veterinary Sciences, University of Messina, 98168 Messina, Italy; mlaragona@unime.it; 3 Institute of Marine Biological Resources and Biotechnology, National Research Council (IRBIM, CNR), 98164 Messina, Italy; spano@unime.it; 4 Department of Clinical and Experimental Medicine, University of Messina, Padiglione C, A. O. U. Policlinico “G. Martino”, 98124 Messina, Italy; angelofumia@gmail.com; 5 Department of Biomedical, Dental and Morphological and Functional Imaging, University of Messina, 98125 Messina, Italy

**Keywords:** comparative immunology, dendritic cells, evolution, immunity, confocal microscopy

## Abstract

**Simple Summary:**

This research aimed to define intestinal DCs from myxines to teleosts by using immunohistochemistry with TLR-2, Langerin/CD207, and MHC II. In particular, we analyzed intestinal DCs in several species of fish phylogenetically organized: *Eptatretus cirrhatus* (myxines), *Scyliorhinus canicula* (Chondrichthyes), *Polypterus senegalus* (Osteichthyes, *Brachiopterygii*), *Lepisosteus oculatus* (Osteichthyes, *Holostei),* and *Clarias batrachus* (Osteichthyes, *Teleostei).* The findings show that intestinal DCs and DC-like cells are positive to all the antibodies tested, demonstrating the presence of these cells phylogenetically from myxines to teleosts. These results could improve the state-of-the-art on the evolution of the immune system and the importance of sentinel cells in the body’s defense. In addition, we demonstrated with immunohistochemistry the presence of MHC II in myxines (*Eptatretus cirrhatus*) for the first time.

**Abstract:**

Dendritic cells (DCs) are antigen-presenting cells (APCs) that regulate the beginning of adaptive immune responses. The mechanisms of tolerance to antigens moving through the digestive tract are known to be regulated by intestinal DCs. Agnatha and Gnathostoma are descendants of a common ancestor. The Ostracoderms gave rise to Cyclostomes, whereas the Placoderms gave rise to Chondrichthyes. *Sarcopterygii* and *Actinopterygii* are two evolutionary lines of bony fishes. *Brachiopterygii* and *Neopterygii* descend from the *Actinopterygii*. From *Neopterygii*, *Holostei* and *Teleostei* evolved. Using immunohistochemistry with TLR-2, Langerin/CD207, and MHC II, this study aimed to characterize intestinal DCs, from myxines to teleosts. The findings reveal that DCs are positive for the antibodies tested, highlighting the presence of DCs and DC-like cells phylogenetically from myxines, for the first time, to teleosts. These findings may aid in improving the level of knowledge about the immune system’s evolution and these sentinel cells, which are crucial to the body’s defense.

## 1. Introduction

Most elements of the innate and adaptive immune system of higher vertebrates, including molecules and receptors, such as immunoglobulins, B-cell receptors (BCRs), Class I and II major histocompatibility complexes (MHC I and MHC II), CD4, CD8, T-cell receptors (TCRs), and dendritic cells (DCs), have been identified in teleosts [1]. Due to their phylogenetic position, teleosts are recognized as an excellent animal model for understanding the evolution of the immune system [2].

DCs are antigen-presenting cells (APCs) that play a key role in regulating the onset of adaptive immune responses. Through the expression of a wide range of recognition receptors, they can quickly detect antigens of a different nature in the mucosal membranes, internalizing and presenting them via MHC I or MHC II to T cells to initiate an adaptive immune response [3,4,5]. Class I and II MHC genes have been identified not only in bony fishes and tetrapods but also in cartilaginous fishes, the most primitive jawed vertebrates [6]. Studies on teleosts have demonstrated the presence of DCs by identifying positive Langerin/CD207 cells [7], cells with DC-like morphology, or cells expressing specific DCs markers [8,9]. The CD207 gene has been detected in bony [7] and cartilaginous fishes [10], and a recent study provided a molecular basis for analyzing the immunological function of DCs in grass carp [11]. Soleto and colleagues in 2019 reported on the identification of DCs in the intestine of rainbow trout, using CD8α^+^ and MHC II [12].

Intestinal DCs are known to regulate the mechanisms of tolerance to antigens passing through the intestinal tract [13]. These cells are part of specialized tissue, called gut-associated lymphoid tissue (GALT), which has immune cells located mostly between the submucosa and mucosa, such as macrophages, DCs, lymphocytes, and granulocytes, while intraepithelial lymphocytes can be found in the mucosa [14,15]. Therefore, these cells play a key role in maintaining the balance between tolerance and immunity in the mucosa. DCs identified in teleosts by expression of CD103, CD141, and Toll-like receptor (TLR)-3 suggested that these cells are potential common ancestors for mammals [12]. TLRs are expressed on DCs; in particular, TLR-2 plays a role in the activation of these cells in fish, as shown by previous studies, both in bony [16,17] and cartilaginous fishes [10]. Cells morphologically similar to DCs have been identified in several fish species, such as *Scyliorhinus canicula* (Linnaeus, 1758) [10], *Oncorhynchus mykiss* (Walbaum, 1792) [18], *Oryzias latipes* (Temminck and Schlegel, 1846) [19], *Salmo salar* (Linnaeus, 1758) [20], and *Periophthalmodon schlosseri* (Pallas, 1770) [21].

**Figure 1:**
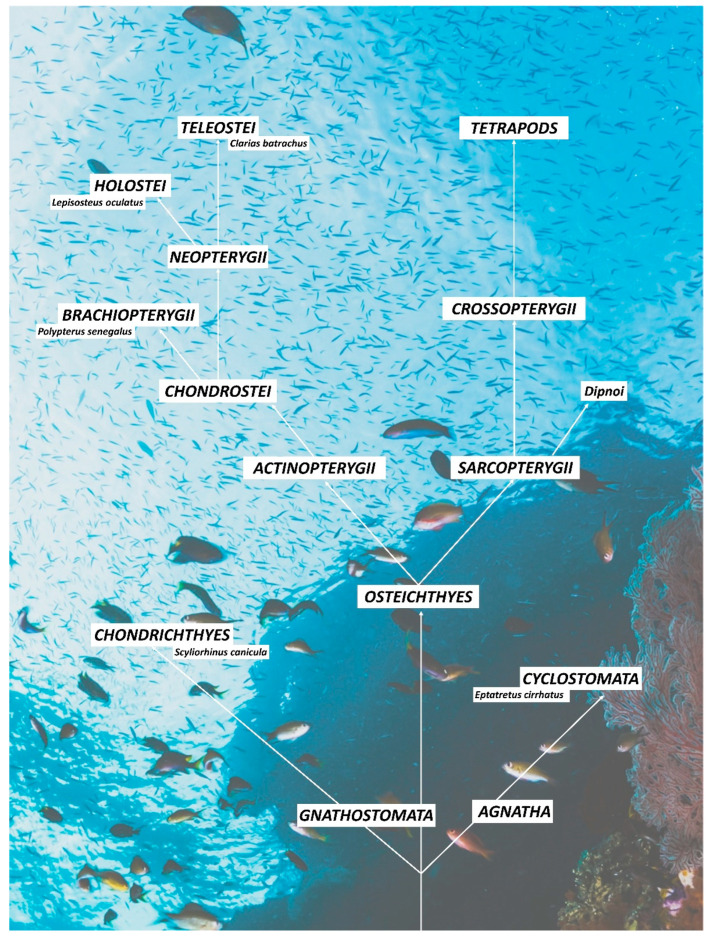
Evolutive relationships among the studied species. Agnatha and Gnathostoma evolved from a common ancestor. Cyclostomes (as the present myxines such as *Eptatretus cirrhatus* Forster, 1801) originated from the Agnatha, while Chondrichthyes (e.g., *Scyliorhinus canicula* Linnaeus, 1758) evolved from the Gnathostomata. The bony fishes also evolved from the Gnathostomata and have given two evolutionary lines: Sarcopterygii and Actinopterygii. The present tetrapods derived from the Sarcopterygii. Chondrostei developed from Actinopterygii. They gave rise to Brachiopterygii and Neopterygii. The former includes the *Polypterus senegalus* Cuvier, 1829. From the latter, the evolved Holostei (e.g., *Lepisosteus oculatus* Winchell, 1864) and Teleostei (e.g., *Clarias batrachus* Linnaeus, 1758).

The actual biodiversity of fish fauna is the result of a long evolutionary history that began over 500 million years ago which saw the emergence and extinction of different groups of fishes until the establishment of the currently dominant group of bony fishes, the teleosts. The first fishes, known as Ostracoderms, did not have mandibles (Agnatha), and their bodies, which were small and generally flattened, were covered by bony plates. The descendants of the Ostracoderms are the current lampreys and myxines. The filter-feeding habits were then abandoned to switch to mechanisms of active predation towards larger organisms, with the appearance of the jaws (Gnathostoma). This latter includes Chondrichthyes, or cartilaginous fishes, derived from Placoderms [22] and Osteichthyes, or bony fishes. The phylogeny of bony fishes has two evolutionary lines, Actinopterygii and Sarcopterygii. From the Sarcopterygii evolved the Crossopterygii, from which the tetrapod originated. The Actinopterygii have given rise to the present great diversity of fish species. The *Polypterus senegalus*, which is the living species with the most primitive characteristics of Actinopterygii [23], has kept functioning lungs, as well as the Dipnoi among the *Sarcopterygii*. The Actinopterygii is divided into the *Chondrostei*, which includes, among others, the sturgeons, and the Neopterygii, which comprises most of the current orders. The Neopterygii includes *Holostei* and *Teleostei*. Of the former, the *Lepisosteiformes* (spotted gar) and the *Amiiformes* (bowfin) still survive. The teleosts represent today the most diversified group of vertebrates, with about 21.000 species adapted to the most disparate environments [24,25,26] (Figure 1).

This study aimed to characterize intestinal DCs, from myxines to teleosts, using immunohistochemistry with TLR-2, Langerin/CD207, and MHC II. By studying DCs in different evolutionary lines of fish, we tried to provide a clearer picture of the phylogenetically conserved characteristics and functions of DCs in vertebrates.

## 2. Materials and Methods

### 2.1. Samples

The gut samples of hagfish *(Eptatretus cirrhatus)*, small-spotted catshark *(Scyliorhinus canicula),* Senegal bichir *(Polypterus senegalus),* spotted gar *(Lepisosteus oculatus),* and walking catfish *(Clarias batrachus)* used in this study were taken from our laboratory’s histotheca and went through the regular procedures for producing durable preparations for optical microscopy and paraffin block storage.

### 2.2. Tissue Preparation

For 12–18 h, samples were fixed in 4% paraformaldehyde in 0.1 M phosphate-buffered saline (pH 7.4), dehydrated in graded ethanol, rinsed in xylene, and embedded in Paraplast^®^ (McCormick Scientific LLC, St. Louis, MO, USA). Finally, using a rotary microtome, serial sections (3–5 m thick) were obtained (LEICA 2065 Supercut) [27].

### 2.3. Histology and Histochemistry

Serial slices were stained with Mallory Trichrome (04-020802 Bio-Optica Milano S.p.A., Milan, Italy) and Alcian Blue pH 2.5-PAS (04-163802 Bio-Optica Milano S.p.A., Milan, Italy) techniques for light microscopic examination.

### 2.4. Immunofluorescence

**Figure 2:**
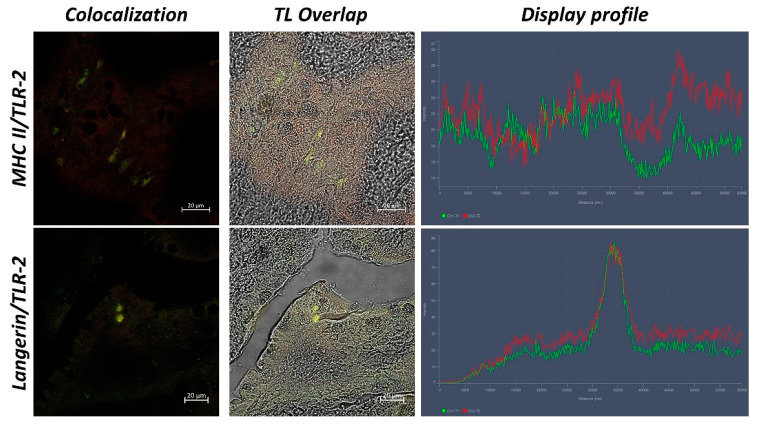
Positive reaction control. Intestine of rat, 40×, scale bars 20 µm. Colocalizations of TLR-2/Langerin/CD207 and TLR-2/MHC II are evident. The “display profile” function confirms the intensity of antibodies colocalization.

Serial sections were deparaffinized and rehydrated numerous times in PBS before being blocked in 2.5% bovine serum albumin (BSA) for 1 h. Sections were then treated with primary antibodies anti-Langerin/CD2017, anti-MHC II, and anti-TLR-2, in a humidified chamber, overnight, at 4 °C, and then utilized individually and in double-label experiments [28,29]. Secondary antisera were Alexa Fluor 594 donkey anti-rabbit IgG TRITC conjugated and Alexa Fluor 488 donkey anti-mouse IgG FITC conjugated (Molecular Probes, Invitrogen, Eugene, OR, USA, 1:300) [30,31]. The sections were mounted with Vectashield (Vector Labs, Burlingame, CA, USA) to prevent photobleaching, and the cover slipped after washing [32]. Negative control tissue preparations were deactivated by removing the primary antibodies. Positive control was carried out on sections of the intestine of rat to confirm the immunopositivity of primary antibodies (Figure 2). Antibodies’ data are summarized in Table 1.

### 2.5. Laser Confocal Immunofluorescence

A Zeiss LSM DUO confocal laser scanning microscope with a META module (Carl Zeiss MicroImaging GmbH, Jena, Germany) equipped with an argon laser (458, 488 l) and two helium–neon lasers was used to examine sections and take images (543 and 633 l) [33]. All the images were digitalized into a 2048 by 2048 pixel array at an 8-bit resolution. Optical slices of fluorescence samples were obtained by using a helium–neon laser (543 nm) and an argon laser (458, 488 nm) with a 1 min, 2 s scanning speed, and up to 8 averages (458 nm) [34]. The photos were processed by using Zen 2011 (LSM 700 Zeiss software, Oberkochen, Germany). Each image was captured quickly to prevent photodegradation. Cropping digital photos and creating the figure montage were performed with Adobe Photoshop CC (Adobe Systems, San Jose, CA, USA) [35]. The intensity profile of an image was shown on a freely selected line by using the laser scanning microscope “display profile” function. The intensity curves are plotted with the scanned images in graphs.

### 2.6. Statistical Analysis

Five sections and ten fields were examined for each sample to acquire data for quantitative analysis. Observation fields were chosen based on the positivity of the cells. ImageJ software was used to evaluate each field [36,37]. After converting the image to 8 bits, a “Threshold” filter and a mask were used to identify cells and remove the background. The cells were then counted by using the “Analyze particles” plug-in. The number of DCs positive to TLR-2, Langerin/CD207, and MHC II for each field was analyzed statistically, using SigmaPlot version 14.0 (Systat Software, San Jose, CA, USA). Data are presented in the form of mean values with standard deviations (SDs). In this sequence, *p*-values less than 0.05 were considered statistically significant: ** *p* ≤ 0.01, and * *p* ≤ 0.05.

### 2.7. Phylogenetic Conservation of Primary Antibodies

MHC is a region of DNA that codes for several proteins involved in the immune response and antigen presentation to T cells [38]. Class II MHC genes in mammals are classified into two categories, classical and non-classical. Class II MHC genes are polymorphic and polygenic and are constitutively expressed in antigen-containing cells such as B cells, macrophages, and DCs [39]. Classical MHC I and II genes are permanently conserved in all jawed vertebrates [40,41]. In addition to classic MHC class II genes, most jawed vertebrates have non-classic MHC class II genes [42]. In particular, the conservation of MHC II sequences from cartilaginous fish to humans is known [43,44,45]. A genetic lineage of MHC II class molecules (MHC II-D) has been identified in bony fishes. In the latter lineage, three subtypes of the MHC II molecule (MHC II-A, -B, and -E) have been identified. Phylogenetic studies of the MHC II molecule genes of subtypes A and B suggest that they may be descended from genes of the subtype A/B, identified in *Lepisosteus oculatus* [46]. The genes of subtype E of the MHC II molecules are present in primitive fish, i.e., in clownfish, sturgeon, and spotted fish (*Lepisosteus oculatus*), as well as in cyprinids (*Cyprinidae*), in the Atlantic salmon (*Salmo salar*), and rainbow trout (*Oncorhynchus mykiss*) [46].

TLRs, found in all classes of vertebrates [47,48,49], are physically and genotypically highly conserved receptors [50,51] that are involved in immune response [52]. Phylogenetically, TLRs—particularly TLR-2—have been characterized in urochordates (tunica and endostyle of ascidian *Styela plicata)* [53,54], cartilaginous fishes [10], bony fishes [55], and other upper vertebrates [48,56]. Sharks are interesting species from an evolutionary point of view, as they are considered the first species to have evolved adaptive immune responses in addition to the innate immune system. One of the components of the highly conserved innate immune system is TLRs. In a study by Anandhakumar (2012), a 270 bp amplicon was amplified by using a degenerate primer strategy corresponding to the Toll/IL-1 (TIR) domain of TLR-2 (GenBank ID: JF792 813). The BLAST analysis revealed a maximum nucleotide identity of 87% and 76% with the TLR-2 of upper mammals and teleosts, respectively [57]. Phylogenetic analysis revealed a clustering of the TIR sequence of sharks closer to humans, cattle, goats, sheep, and birds than other fish species [57]. By comparing the TLR profiles of two species of fish (*Danio rerio* Hamilton, 1822; and *Takifugu rubripes* Temminck and Schlegel, 1850), a collection of orthologous genes with significant sequence conservation in human TLRs was discovered [58,59].

Langerin/CD207 is a type C lectin that is detectable in DCs in most epithelial and connective tissues [60]. Studies have shown that the expression of markers such as MHC II [61,62] and Langerin/CD207 is highly conserved among vertebrates [7]. CD207 has been proposed as a potential marker of DCs in *Scyliorhinus canicula* [10]. In the study, the authors demonstrated the presence of DC-like cells in the gut-associated lymphoid tissue (GALT), using an antibody panel composed of Langerin/CD207, TLR-2, and S-100. Lovy et al., 2009 characterized DCs in the hematopoietic organs of salmonids. Specimens of Atlantic salmon (*Salmo salar*) and rainbow trout (*Oncorhynchus mykiss*) were used for the research. Incubation of the spleen with CD207 revealed positive Langerin/CD207 cells [7]. The cephalic kidney of Atlantic salmon also showed a positive reaction with CD207. These data suggest that Langerin/CD207 is a potential marker for DCs in primitive vertebrates [7]. Kordon et al., 2016 researched DCs in *Ictalurus punctatus*, the catfish. Using antibodies to Langerin/CD207, DC-like cells were labeled in the spleen and cephalic kidney of catfish [63]. Several studies have shown the presence of similar DCs in zebrafish [9,64]. Lugo-Villarino et al., 2010 identified morphologically similar cells to mammalian DCs in *Danio rerio*, demonstrating that the cellular constituents of the antigen presentation process appear well preserved in different species, from teleosts to upper vertebrates [9].

## 3. Results

### 3.1. Histological and Histochemical Description

**Figure 3:**
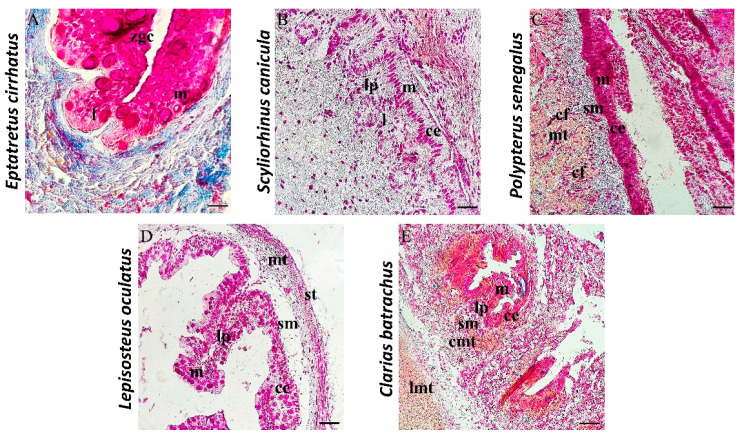
Sections of intestine stained with Mallory Trichrome, 40×, scale bars 40 µm. (**A**) Mucosa (m), submucosa (sm), and Muscolaris serosa (ms) are the three layers of the gut of *Eptatretus cirrhatus*. The mucosa is arranged in zigzag ridges, with evident zimogenous cells (zgc), and scattered immune cells (l). (**B**) The intestine of *Scyliorhinus canicula* is separated into mucosa, submucosa, muscolaris, and serosa. The mucosa is organized in folds that are virtually obliquely oriented. A columnar epithelium (ce) of enterocytes forms the mucous layer (m). Scattered immune cells (l) are evident in lamina propria (lp). (**C**) The gut of *Polypterus senegalus* presents a mucosa (m) with columnar epithelium (ce), submucosa (sm), muscolaris (mt), and serosa (st). The mucous layer of the intestine shows goblet cells and columnar epithelial cells (ce). The thickness of the submucosa contains collagen fibers (cfs). (**D**) There are four strata in the gut of *Lepisosteus oculatus*: mucosa (m), submucosa (sm), muscolaris (st), and serosa (st). In the mucosa, longitudinal folds and a columnar epithelium (ce) accompanied by goblet cells can be seen. (**E**) The intestine of *Clarias batrachus* morphologically shows four laminae: mucosa (m), submucosa (sm), muscolaris (mt), and serosa (st). The mucosa of the intestine is folded and has a columnar epithelium (ce) comprising enterocytes and mucipar caliciform cells. Muscularis is organized in longitudinal (lmt) and circular (cmt).

**Figure 4:**
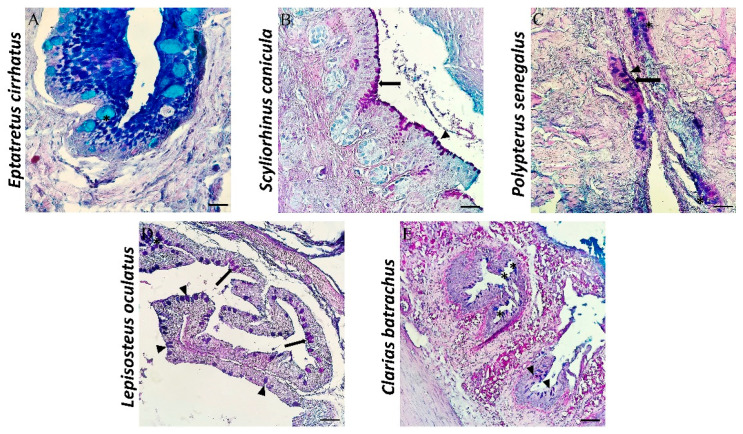
Sections of intestine stained with AB/PAS, 40×, scale bar 40 µm. (**A**) In the intestine of *Eptatretus cirrhatus*, mucous cells according to the type of secretion (acid in blue, neutral in magenta, and mixed in purple) can be distinguished. Staining highlights several mucous cells with an ovoid shape in blue (*) with acidophilic secretion. (**B**) Two mucous cell populations, neutral mucous (magenta) (arrows) and mixed secretion mucous cells (neutral and acid) (purple) (arrowheads), were detected in the intestine of *Scyliorhinus canicula*. (**C**) Mucous cells are abundant and can take different forms, namely ovoid, columnar, or pear-shaped, in the intestine of *Polypterus senegalus*. The presence of all three types of mucins (acidic, neutral, and mixed) is evident. (**D**) In sections of the intestine of *Lepisosteus oculatus*, the mucous secretion shows the presence of acidic, neutral, and mixed mucins. (**E**) The mucous cells on the apex of the intestinal crests of *Clarias batrachus* are strongly positive for AB/PAS histochemical staining, demonstrating the presence of acidic, neutral, and mixed mucins.

All the examined intestines show the subdivision into four layers, which, from the inside out, are mucosa, submucosa, muscularis, and serosa, except for the intestine of *Eptatretus*. The intestine of the latter shows a subdivision into three layers: mucosa, submucosa, and muscularis serosa. Mallory histological staining shows a mucosa organized in permanent and wide zigzag ridges. Zimogenous cells (zgcs) are evident and useful for the digestion process. In the mucosa, there are also scattered immune cells (l) (Figure 3A). AB/PAS histochemical staining used to distinguish mucous cells according to the type of secretion (acid in blue, neutral in magenta, and mixed in purple) highlights several mucous cells with an ovoid shape in blue, with acidophilic secretion (Figure 4A).

The mucosa of *Scyliorhinus canicula* is organized in folds with an almost oblique orientation and a conformation comparable to that of the intestinal villi of mammals. The mucous layer is characterized by a columnar epithelium of enterocytes (Figure 3B), with many PAS-positive mucous cells (Figure 4B). Two mucous cell populations, neutral mucous (magenta) and mixed secretion mucous cells (neutral and acid) (purple), were detected with AB/PAS histochemical staining (Figure 4B). Scattered immune cells (l) forming intestinal-associated lymphoid tissue (GALT) in the lamina propria (lp) are evident.

The mucosa of *Polypterus senegalus* shows a columnar epithelium, with different types of cells that make up the mucous layer of the intestine: PAS-positive mucous cells and columnar epithelial cells (Figure 3C). Goblet cells are abundant and can take different forms, i.e., ovoid, columnar, or pear-shaped (Figure 4C). Collagen fibers are present in the thickness of the submucosa (Figure 3C). AB/PAS coloration shows the presence of all three types of mucins: acidic, neutral, and mixed (Figure 4C).

In the intestine of *Lepisosteus oculatus,* longitudinal folds and a columnar epithelium associated with goblet cells in the mucous layer are observed (Figure 3D). The mucous secretion appears to be different, showing the presence of acidic, neutral, and mixed mucins (Figure 4D).

Morphologically, the intestine of *Clarias batrachus* shows an intestinal mucosa organized in folds and characterized by a columnar epithelium of enterocytes and goblet cells. The lamina propria is made up of loose connective tissue and penetrates inside the ridges, forming their central axis (Figure 3E). The mucous cells on the apex of the intestinal crests are strongly positive for AB/PAS histochemical staining, demonstrating the presence of acidic, neutral, and mixed mucins (Figure 4E).

### 3.2. Confocal Scanning laser Microscopy

**Figure 5:**
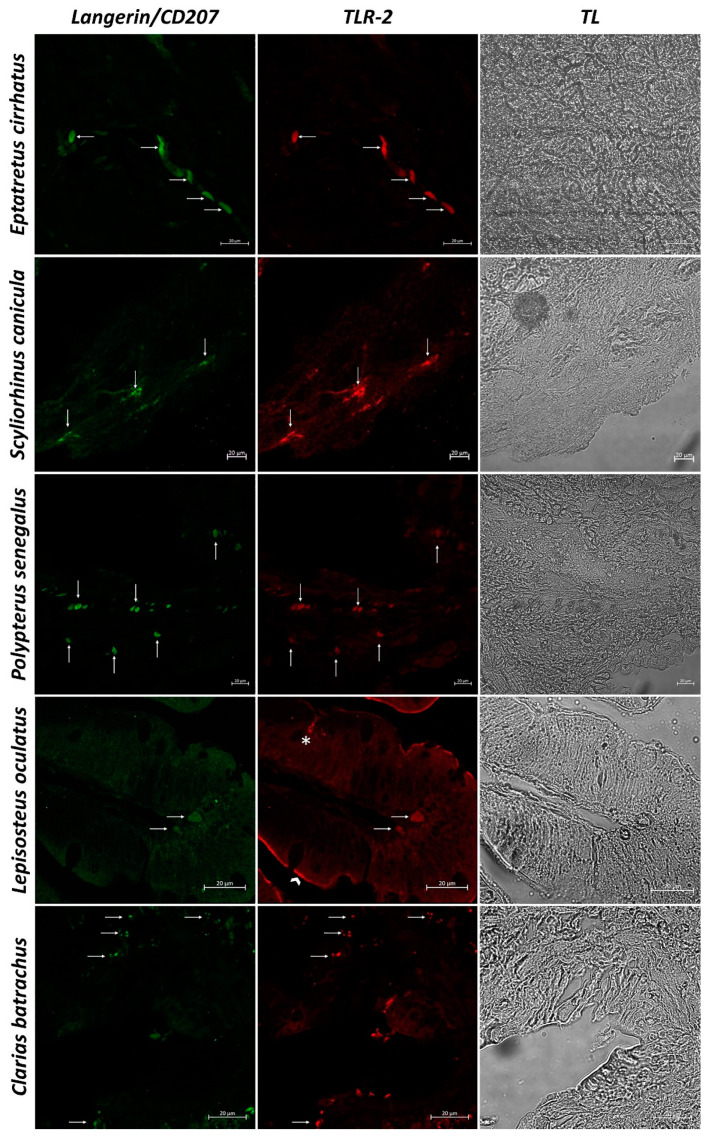
Immunofluorescence of Langerin/CD207 and TLR-2, 40×, scale bar 20 µm. The immunopositivity to Langerin/CD207 (green) and TLR-2 (red) is evident in the mucosa (*Polypterus senegalus*, *Lepisosteus oculatus*, and *Clarias batrachus*) and submucosa (*Eptatretus cirrhatus*, *Scyliorhinus canicula*, and *Clarias batrachus*) (arrows). Some scattered immune cells (*) TLR-2 positive in the gut of *Lepisosteus oculatus* is detected. Enterocytes (arrowhead) are positive for TLR-2 in the intestine of *Lepisosteus oculatus*. Transmitted Light (TL) is used to compare the organ morphology.

**Figure 6:**
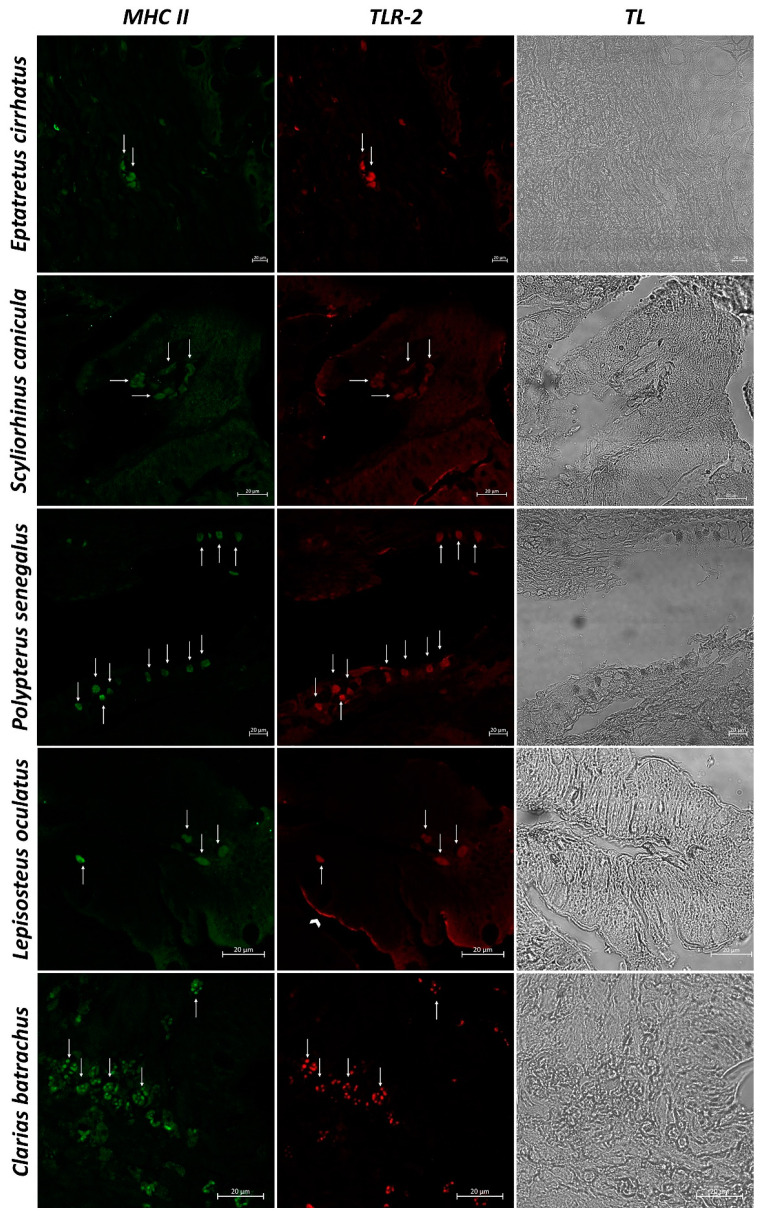
Immunofluorescence of MHC II and TLR-2, 40×, scale bar 20 µm. The immunopositivity to MHC II (green) and TLR-2 (red) is evident in the mucosa (*Polypterus senegalus, Lepisosteus oculatus,* and *Clarias batrachus*), lamina propria (*Scyliorhinus canicula*), and submucosa (*Eptatretus cirrhatus*) (arrows). Enterocytes (arrowhead) are positive for TLR-2 in the intestine of Lepisosteus. Transmitted Light (TL) is used to compare the organ morphology.

**Figure 7:**
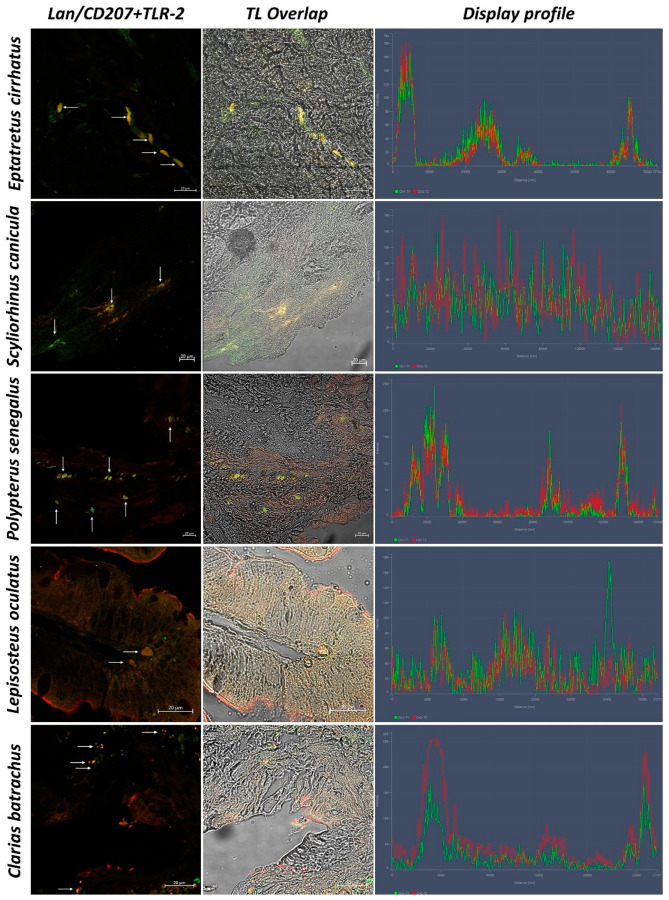
Colocalization of Langerin/CD207 and TLR-2, 40×, scale bar 20 µm. The colocalization of Langerin (green) and TLR-2 (red) is evident (arrows). Overlap in Transmitted Light (TL) is used to compare the organ morphology. The “display profile” function was used to confirm the antibodies colocalization.

**Figure 8:**
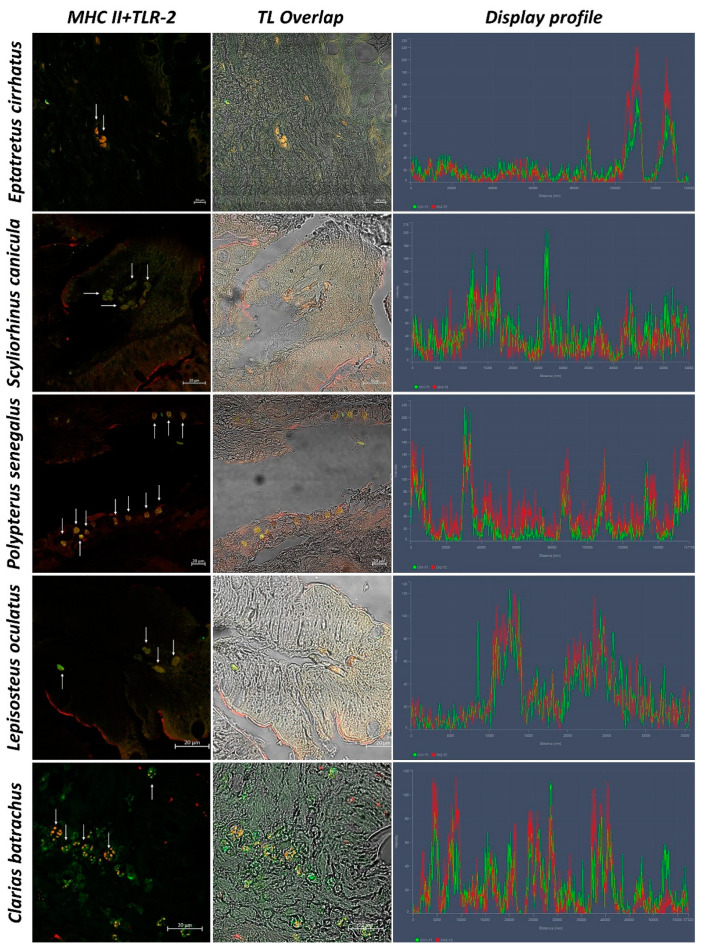
Colocalization of MHC II and TLR-2, 40×, scale bar 20 µm. The colocalization of MHC II (green) and TLR-2 (red) is evident (arrows). Overlap in Transmitted Light (TL) is used to compare the organ morphology. The “display profile” function was used to confirm the antibodies’ colocalization.

In sections of intestinal tissue of *Eptatretus cirrhatus*, DC-like cells positive to Langerin/CD207, MHC II, and TLR-2 are evident in the submucosa (Figure 5 and Figure 6). *Scyliorhinus canicula* has similar DCs, which are positive for the antibodies tested in the submucosa. Positive DC-like cells to MHC II/TLR-2 in the lamina propria are evident (Figure 5 and Figure 6). Sections of the intestine of *Polypterus senegalus* show the immunoreactivity of Langerin/CD207, MHC II, and TLR-2 positive cells in the mucosa (Figure 5 and Figure 7). Immunopositivity to the same antibodies is evident in the mucosa of the intestine of *Lepisosteus oculatus* (Figure 5 and Figure 6). Langerin/CD207, MHC II, and TLR-2 positive DCs are also identified in the mucosa and submucosa of the intestine of *Clarias batrachus* (Figure 5 and Figure 6). In all tissues, there is strong colocalization, as also confirmed by the graphs obtained with the “display profile” function by confocal microscope (Figure 7 and Figure 8).

### 3.3. Statistical Analysis

The statistical analysis shows an increasing number of DC-like cells from *Eptatretus cirrhatus* to *Clarias batrachus*. The number of DC-like cells is inferior in the intestine of *Eptatretus cirrhatus,* the most primitive fish of this study, while *Clarias batrachus*, as the most evolved, presents the higher number of those cells. Data are reported in Table 2.

## 4. Discussion

For hundreds of millions of years, the immune system has diversified and evolved into a highly complex set of cells and molecular mechanisms for the defense and regeneration of the body. Jawless vertebrates, such as myxines and lampreys, were the first to link innate responses with some adaptive elements [65,66]. Whereas all jawed vertebrates, from elasmobranchs to mammals, are endowed with innate and adaptive immunity [67]. In general, innate immunity is a rapid and non-specific response associated with the presence of humoral and cellular elements. In contrast, adaptive immunity uses the induction of specialized cells [68].

DCs are APCs and are able to orchestrate the immune response of T lymphocytes, so they are indispensable immune sentinels. They come from progenitors in the bone marrow through hematopoiesis, a highly regulated developmental process involving multiple cellular and molecular events [69]. Cells with the morphology of DCs are found in all gnathostomes, and they have a high expression of MHC class II molecules. These cells are highly conserved and have been characterized in different species, from cartilaginous fish to mammals [70]. Specifically, DC-like cells have been identified in sharks, bony fish, amphibians, reptiles, birds, and mammals [71]. DCs had not been characterized in the Agnatha and the amphioxus; however, paralogous genes of the MHC complex have been identified in the amphioxus, as well as two populations of lymphocyte-like cells [72]. The common phenotype is characterized by the expression of S100 antigen [73], MHC II, CD8a [74], CD83, CD86, Langerin/CD207 [63], and others, including TLRs, highly conserved molecules [21,75].

Our study has characterized intestinal DCs, from myxines to teleosts. Generally, the intestine of fishes consists of several layers: mucous, submucous, muscular, and serosa. The mucous layer contains goblet cells, which are essential for the lubrication, absorption, and transport of macromolecules; the increase of digestive capacity; and the epithelial prevention of acidity. Gastrointestinal mucus is made up of different types of mucous substances or mucins, including neutral mucin and acid mucin [76]. The intestine and associated lymphoid tissue (GALT) of jawed fishes play a significant role in fish immunology. Pathogens are recognized not only by specialized APCs but also by epithelial cells that form the primary cell barrier. There is a strong relationship between epithelial cells and T cells, which are important in intestinal immunity [10].

The intestine of the myxines probably derives from the microphage feeding of ancestral craniates. They do not possess a defined stomach or a differentiated intestine into small and large, so digestion takes place directly in the rectilinear intestinal tract through lipases. Histologically, the intestine of myxines is characterized by three layers: mucous, submucous, and muscularis serosa [77].

Histologically, our results are in agreement with several studies [78,79,80], showing in the analyzed intestines the presence of a consistent mucosa, organized in crests, with numerous goblet cells and well-defined enterocytes, a submucosa, a muscularis, and a serosa. AB/PAS histochemical staining also allowed us to identify different mucopolysaccharide components, especially acidic mucins in blue and neutral mucins in magenta, as already reported in a previous article [81].

Our study characterized DCs in fish by using anti-TLR-2, MHC II, and Langerin/CD207 antibodies. We identified, for the first time, immunopositive cells for TLR-2, MHC II, and Langerin/CD207 in the intestine of *Eptatretus cirrhatus*. Using the “display profile” function, we confirmed the colocalization of TLR-2/MHC II and TLR-2/Langerin/CD207.

In Agnatha, seven TLRs have been identified, while mammals have 13 recognized TLRs [82]. These antibodies, as mentioned above, are highly expressed on the surface of DCs. These data suggest the presence of DCs-like in the myxines. Further studies would be useful to confirm our data on the presence of these antibodies in this Agnatha and could provide additional information on the phylogenesis of the immune system. DC-like cells were also found in *Scyliorhinus canicula*, according to a previous study by Lauriano et al. [10], where DC-like cells that are immunoreactive to TLR-2 and Langerin/CD207 were identified. Furthermore, several studies have also shown the presence of DC-like MHC-II-positive cells in Chondrichthyes [70,83].

In sections of the intestine of *Polypterus senegalus*, DC-like cells that are immunopositive to the antibodies tested appear evident, with marked colocalization, as confirmed by the “display profile” function. These data suggest, for the first time, the hypothesis of the presence of these cells in Brachiopterygii.

Moreover, our results show positive cells to the antibodies tested also in the intestine of *Lepisosteus oculatus*. According to a study by Dong et al., 2021 on *Paralycthis olivaceus*, a fish belonging to the order of pleuronectiforms, subclass Neopterygii, which characterized DCs cells with CD83 and MHC II, show MHC II immunopositive DCs [84]. Biraj Sharma et al., 2018 demonstrated the presence of TLR-2 in Neopterygii [85].

Finally, we characterized DCs in the intestine of teleost *Clarias batrachus*. Previous studies have demonstrated the presence of classical DCs in teleosts, characterizing immunoreactivity to MHC II [18,74,86], and Langerin/CD207 [7], supporting our data. A study by Soleto et al., 2019 characterized DCs in rainbow trout (*Oncorhynchus mykiss*) with MHC II and identified the presence of several TLRs, such as TLR-2 and TLR-3, thus further confirming our results on the intestine of *Clarias batrachus* [12].

Our study showed the presence of DCs and DC-like cells phylogenetically, from myxines, for the first time, to teleosts. These data may help to improve the state-of-the-art of information on the evolution of the immune system and on these sentinel cells, which are essential for the body’s defense. Moreover, we demonstrated, with immunohistochemistry, the presence of MHC II in *Eptatretus cirrhatus*, thus improving the knowledge about these peculiar immune molecules.

## Figures and Tables

**Table 1:** Antibodies data.

Antibody	Supplier	Dilution	Animal Source
Langerin/CD207	Santa Cruz Biotechnology, Inc., Dallas, TX, USA	1:250	Mouse
MHC II	Santa Cruz Biotechnology, Inc., Dallas, TX, USA	1:250	Mouse
TLR-2	Active Motif, La Hulpe, Belgium; Europe	1:125	Rabbit
Alexa Fluor 488 donkey anti-mouse IgG FITC conjugated	Molecular Probes, Invitrogen	1:300	Mouse
Alexa Fluor 594 donkey anti-rabbit IgG TRITC conjugated	Molecular Probes, Invitrogen	1:300	Rabbit

**Table 2:** Statistical analysis data (mean values with SD).

	Lan/CD207	TLR-2	Lan/CD207 + TLR-2	MHC II	TLR-2	MHC II + TLR-2
*Eptatretus cirrhatus*	116.30 ± 18.17 *	152.70 ± 33.59 *	107.71 ± 13.80 *	90.34 ± 9.65 **	146.20 ± 11.63 **	76.41 ± 9.39 *
*Scyliorhinus canicula*	145.29 ± 11.37 *	175.17 ± 19.18 **	138.56 ± 27.56 **	168.12 ± 7.78 **	176.40 ± 11.57 *	152.17 ± 11.48 **
*Polypterus senegalus*	203.80 ± 14.08 *	231.10 ± 24.06 *	193.43 ± 10.68 *	220.75 ± 7.46 *	251.01 ± 8.97 *	205.72 ± 12.46 *
*Lepisosteus oculatus*	172.72 ± 20.72 **	222.43 ± 17.99 *	166.11 ± 14.44 **	217.58 ± 11.56 *	238.29 ± 10.86 *	212.10 ± 10.18 **
*Clarias batrachus*	226.08 ± 16.85 *	249.98 ± 18.93 *	215.38 ± 11.72 *	237.59 ± 22.36 *	261.43 ± 10.40 *	221.67 ± 9.42 *

* *p* ≤ 0.05; ** *p* ≤ 0.01.

## Data Availability

Not applicable.

## References

[1] Wu L., Qin Z., Liu H., Lin L., Ye J., Li J. (2020). Recent Advances on Phagocytic B Cells in Teleost Fish. Front. Immunol..

[2] Sunyer J.O. (2013). Fishing for Mammalian Paradigms in the Teleost Immune System. Nat. Immunol..

[3] Mellman I., Steinman R.M. (2001). Dendritic Cells. Cell.

[4] Johnstone C., Chaves-Pozo E. (2022). Antigen Presentation and Autophagy in Teleost Adaptive Immunity. IJMS.

[5] Alesci A., Pergolizzi S., Fumia A., Miller A., Cernigliaro C., Zaccone M., Salamone V., Mastrantonio E., Gangemi S., Pioggia G. (2022). Immune System and Psychological State of Pregnant Women during COVID-19 Pandemic: Are Micronutrients Able to Support Pregnancy?. Nutrients.

[6] Okamura K., Dijkstra J.M., Tsukamoto K., Grimholt U., Wiegertjes G.F., Kondow A., Yamaguchi H., Hashimoto K. (2021). Discovery of an Ancient MHC Category with Both Class I and Class II Features. Proc. Natl. Acad. Sci. USA.

[7] Lovy J., Savidant G.P., Speare D.J., Wright G.M. (2009). Langerin/CD207 Positive Dendritic-like Cells in the Haemopoietic Tissues of Salmonids. Fish Shellfish Immunol..

[8] Johansson P., Corripio-Miyar Y., Wang T., Collet B., Secombes C.J., Zou J. (2012). Characterisation and Expression Analysis of the Rainbow Trout (*Oncorhynchus mykiss*) Homologue of the Human Dendritic Cell Marker CD208/Lysosomal Associated Membrane Protein 3. Dev. Comp. Immunol..

[9] Lugo-Villarino G., Balla K.M., Stachura D.L., Bañuelos K., Werneck M.B.F., Traver D. (2010). Identification of Dendritic Antigen-Presenting Cells in the Zebrafish. Proc. Natl. Acad. Sci. USA.

[10] Lauriano E.R., Pergolizzi S., Aragona M., Montalbano G., Guerrera M.C., Crupi R., Faggio C., Capillo G. (2019). Intestinal Immunity of Dogfish Scyliorhinus Canicula Spiral Valve: A Histochemical, Immunohistochemical and Confocal Study. Fish Shellfish Immunol..

[11] Wang H., Chen X., Li S., Zhou C., Xu L., Wu Z., Chen X. (2021). Identification and Expression Analysis of Langerhans Cells Marker Langerin/CD207 in Grass Carp, Ctenopharyngodon Idella. Gene.

[12] Soleto I., Granja A.G., Simón R., Morel E., Díaz-Rosales P., Tafalla C. (2019). Identification of CD8α+ Dendritic Cells in Rainbow Trout (*Oncorhynchus mykiss*) Intestine. Fish Shellfish Immunol..

[13] Persson E.K., Scott C.L., Mowat A.M., Agace W.W. (2013). Dendritic Cell Subsets in the Intestinal Lamina Propria: Ontogeny and Function: HIGHLIGHTS. Eur. J. Immunol..

[14] Alesci A., Fumia A., Miller A., Calabrò C., Santini A., Cicero N., Lo Cascio P. (2022). Spirulina Promotes Macrophages Aggregation in Zebrafish (*Danio rerio*) Liver. Nat. Prod. Res..

[15] Estensoro I., Mulero I., Redondo M.J., Alvarez-Pellitero P., Mulero V., Sitja-Bobadilla A. (2014). Modulation of Leukocytic Populations of Gilthead Sea Bream (*Sparus aurata*) by the Intestinal Parasite *Enteromyxum leei* (Myxozoa: Myxosporea). Parasitology.

[16] Volarevic V., Markovic B.S., Jankovic M.G., Djokovic B., Jovicic N., Harrell C.R., Fellabaum C., Djonov V., Arsenijevic N., Lukic M.L. (2019). Galectin 3 Protects from Cisplatin-Induced Acute Kidney Injury by Promoting TLR-2-Dependent Activation of IDO1/Kynurenine Pathway in Renal DCs. Theranostics.

[17] Dahiya P., Hussain M.A., Mazumder S. (2021). MtROS Induced via TLR-2-SOCE Signaling Plays Proapoptotic and Bactericidal Role in Mycobacterium Fortuitum-Infected Head Kidney Macrophages of Clarias Gariepinus. Front. Immunol..

[18] Bassity E., Clark T.G. (2012). Functional Identification of Dendritic Cells in the Teleost Model, Rainbow Trout (*Oncorhynchus mykiss*). PLoS ONE.

[19] Aghaallaei N., Bajoghli B., Schwarz H., Schorpp M., Boehm T. (2010). Characterization of Mononuclear Phagocytic Cells in Medaka Fish Transgenic for a *Cxcr3a:Gfp* Reporter. Proc. Natl. Acad. Sci. USA.

[20] Haugland G.T., Jordal A.-E.O., Wergeland H.I. (2012). Characterization of Small, Mononuclear Blood Cells from Salmon Having High Phagocytic Capacity and Ability to Differentiate into Dendritic like Cells. PLoS ONE.

[21] Lauriano E.R., Faggio C., Capillo G., Spanò N., Kuciel M., Aragona M., Pergolizzi S. (2018). Immunohistochemical Characterization of Epidermal Dendritic-like Cells in Giant Mudskipper, Periophthalmodon Schlosseri. Fish Shellfish Immunol..

[22] Zhu Y., Giles S., Young G.C., Hu Y., Bazzi M., Ahlberg P.E., Zhu M., Lu J. (2021). Endocast and Bony Labyrinth of a Devonian “Placoderm” Challenges Stem Gnathostome Phylogeny. Curr. Biol..

[23] Zaccone D., Icardo J.M., Kuciel M., Alesci A., Pergolizzi S., Satora L., Lauriano E.R., Zaccone G. (2017). Polymorphous Granular Cells in the Lung of the Primitive Fish, the Bichir P Olypterus Senegalus. Acta Zool..

[24] Kent G.C. (1997). Anatomia Comparata dei Vertebrati.

[25] Liem K.F., Bemis W.E., Grande L. (2012). Anatomia Comparata dei Vertebrati: Una Visione Funzionale ed Evolutiva.

[26] Giavini E., Menegola E. (2010). Manuale di Anatomia Comparata.

[27] Monforte M.T., Smeriglio A., Germanò M.P., Pergolizzi S., Circosta C., Galati E.M. (2018). Evaluation of Antioxidant, Antiinflammatory, and Gastroprotective Properties of *Rubus fruticosus* L. Fruit Juice: Biological Properties of *R. fruticosus* L. Fruit Juice. Phytother. Res..

[28] Pergolizzi S., Rizzo G., Favaloro A., Alesci A., Pallio S., Melita G., Cutroneo G., Lauriano E.R. (2021). Expression of VAChT and 5-HT in Ulcerative Colitis Dendritic Cells. Acta Histochem..

[29] Lauriano E.R., Pergolizzi S., Gangemi J., Kuciel M., Capillo G., Aragona M., Faggio C. (2017). Immunohistochemical Colocalization of G Protein Alpha Subunits and 5-HT in the Rectal Gland of the Cartilaginous Fish *Scyliorhinus canicula*. Microsc. Res. Tech..

[30] Pergolizzi S., Alesci A., Centofanti A., Aragona M., Pallio S., Magaudda L., Cutroneo G., Lauriano E.R. (2022). Role of Serotonin in the Maintenance of Inflammatory State in Crohn’s Disease. Biomedicines.

[31] Icardo J.M., Colvee E., Lauriano E.R., Capillo G., Guerrera M.C., Zaccone G. (2015). The Structure of the Gas Bladder of the Spotted Gar, Lepisosteus Oculatus: The Gas Bladder of *Lepisosteus oculatus*. J. Morphol..

[32] Zaccone G., Lauriano E.R., Capillo G., Kuciel M. (2018). Air- Breathing in Fish: Air- Breathing Organs and Control of Respiration. Acta Histochem..

[33] Zaccone G., Fudge D.S., Winegard T.M., Capillo G., Kuciel M., Funakoshi K., Lauriano E.R. (2015). Confocal Imaging and Phylogenetic Considerations of the Subcutaneous Neurons in the Atlantic Hagfish *Myxine glutinosa*. Acta Zool..

[34] Kuciel M., Rita Lauriano E., Silvestri G., Żuwała K., Pergolizzi S., Zaccone D. (2014). The Structural Organization and Immunohistochemistry of G-Protein Alpha Subunits in the Olfactory System of the Air-Breathing Mudskipper, Periophthalmus Barbarus (Linnaeus, 1766) (Gobiidae, Oxudercinae). Acta Histochem..

[35] Lauriano E.R., Żuwała K., Kuciel M., Budzik K.A., Capillo G., Alesci A., Pergolizzi S., Dugo G., Zaccone G. (2016). Confocal Immunohistochemistry of the Dermal Glands and Evolutionary Considerations in the Caecilian, *Typhlonectes natans* (Amphibia: Gymnophiona). Acta Zool..

[36] Schneider C.A., Rasband W.S., Eliceiri K.W. (2012). NIH Image to ImageJ: 25 Years of Image Analysis. Nat. Methods.

[37] D’Iglio C., Natale S., Albano M., Savoca S., Famulari S., Gervasi C., Lanteri G., Panarello G., Spanò N., Capillo G. (2021). Otolith Analyses Highlight Morpho-Functional Differences of Three Species of Mullet (Mugilidae) from Transitional Water. Sustainability.

[38] Bartl S. (1998). What Sharks Can Tell Us about the Evolution of MHC Genes. Immunol. Rev..

[39] Grimholt U. (2016). MHC and Evolution in Teleosts. Biology.

[40] Dijkstra J.M., Yamaguchi T., Grimholt U. (2018). Conservation of Sequence Motifs Suggests That the Nonclassical MHC Class I Lineages CD1/PROCR and UT Were Established before the Emergence of Tetrapod Species. Immunogenetics.

[41] Flajnik M., Ohta Y., Namikawa-Yomada C., Nonaka M. (1999). Insight into the Primordial MHC from Studies in Ectothermic Vertebrates. Immunol. Rev..

[42] Nonaka M., Nonaka M.I. (2016). The Evolution of Major Histocompatibility Complex in Teleosts. The Evolution of the Immune System.

[43] Dijkstra J.M., Yamaguchi T. (2019). Ancient Features of the MHC Class II Presentation Pathway, and a Model for the Possible Origin of MHC Molecules. Immunogenetics.

[44] Smith N.C., Rise M.L., Christian S.L. (2019). A Comparison of the Innate and Adaptive Immune Systems in Cartilaginous Fish, Ray-Finned Fish, and Lobe-Finned Fish. Front. Immunol..

[45] Dooley H., Cooper E.L. (2018). Chondrichthyes: The Immune System of Cartilaginous Fishes. Advances in Comparative Immunology.

[46] Stosik M., Tokarz-Deptuła B., Deptuła W. (2020). Major Histocompatibility Complex in Osteichthyes. J. Vet. Res..

[47] Lauriano E.R., Pergolizzi S., Capillo G., Kuciel M., Alesci A., Faggio C. (2016). Immunohistochemical Characterization of Toll-like Receptor 2 in Gut Epithelial Cells and Macrophages of Goldfish Carassius Auratus Fed with a High-Cholesterol Diet. Fish Shellfish Immunol..

[48] Marino A., Pergolizzi S., Lauriano E.R., Santoro G., Spataro F., Cimino F., Speciale A., Nostro A., Bisignano G. (2015). TLR2 Activation in Corneal Stromal Cells by *Staphylococcus aureus* -Induced Keratitis. APMIS.

[49] Marino A., Santoro G., Spataro F., Lauriano E.R., Pergolizzi S., Cimino F., Speciale A., Nostro A., Bisignano G., Dugo G. (2013). Resveratrol Role in *Staphylococcus aureus* -Induced Corneal Inflammation. Pathog. Dis..

[50] Alesci A., Pergolizzi S., Fumia A., Calabrò C., Lo Cascio P., Lauriano E.R. (2022). Mast Cells in Goldfish (*Carassius auratus*) Gut: Immunohistochemical Characterization. Acta Zool..

[51] Alesci A., Lauriano E.R., Aragona M., Capillo G., Pergolizzi S. (2020). Marking Vertebrates Langerhans Cells, from Fish to Mammals. Acta Histochem..

[52] Alesci A., Fumia A., Lo Cascio P., Miller A., Cicero N. (2021). Immunostimulant and Antidepressant Effect of Natural Compounds in the Management of COVID-19 Symptoms. J. Am. Coll. Nutr..

[53] Alesci A., Pergolizzi S., Lo Cascio P., Capillo G., Lauriano E.R. (2022). Localization of Vasoactive Intestinal Peptide and Toll-like Receptor 2 Immunoreactive Cells in Endostyle of Urochordate *Styela plicata* (Lesueur, 1823). Microsc. Res. Tech..

[54] Lauriano E.R., Aragona M., Alesci A., Lo Cascio P., Pergolizzi S. (2021). Toll-like Receptor 2 and α-Smooth Muscle Actin Expressed in the Tunica of a Urochordate, Styela Plicata. Tissue Cell.

[55] Alesci A., Pergolizzi S., Capillo G., Cascio P.L., Lauriano E.R. (2022). Rodlet Cells in Kidney of Goldfish (*Carassius auratus*, Linnaeus 1758): A Light and Confocal Microscopy Study. Acta Histochem..

[56] Lauriano E.R., Silvestri G., Kuciel M., Żuwała K., Zaccone D., Palombieri D., Alesci A., Pergolizzi S. (2014). Immunohistochemical Localization of Toll-like Receptor 2 in Skin Langerhans’ Cells of Striped Dolphin (*Stenella coeruleoalba*). Tissue Cell.

[57] Anandhakumar C., Lavanya V., Pradheepa G., Tirumurugaan K.G., Dhinakar Raj G., Raja A., Pazhanivel N., Balachandran C. (2012). Expression Profile of Toll-like Receptor 2 MRNA in Selected Tissues of Shark (*Chiloscyllium* sp.). Fish Shellfish Immunol..

[58] Jault C., Pichon L., Chluba J. (2004). Toll-like Receptor Gene Family and TIR-Domain Adapters in Danio Rerio. Mol. Immunol..

[59] Oshiumi H., Matsumoto M., Funami K., Akazawa T., Seya T. (2003). TICAM-1, an Adaptor Molecule That Participates in Toll-like Receptor 3–Mediated Interferon-β Induction. Nat. Immunol..

[60] Mayer W.J., Irschick U.M., Moser P., Wurm M., Huemer H.P., Romani N., Irschick E.U. (2007). Characterization of Antigen-Presenting Cells in Fresh and Cultured Human Corneas Using Novel Dendritic Cell Markers. Investig. Ophthalmol. Vis. Sci..

[61] Morrison R.N., Koppang E.O., Hordvik I., Nowak B.F. (2006). MHC Class II+ Cells in the Gills of Atlantic Salmon (*Salmo salar* L.) Affected by Amoebic Gill Disease. Vet. Immunol. Immunopathol..

[62] Olsen M.M., Kania P.W., Heinecke R.D., Skjoedt K., Rasmussen K.J., Buchmann K. (2011). Cellular and Humoral Factors Involved in the Response of Rainbow Trout Gills to Ichthyophthirius Multifiliis Infections: Molecular and Immunohistochemical Studies. Fish Shellfish Immunol..

[63] Kordon A.O., Scott M.A., Ibrahim I., Abdelhamed H., Ahmed H., Baumgartner W., Karsi A., Pinchuk L.M. (2016). Identification of Langerhans-like Cells in the Immunocompetent Tissues of Channel Catfish, Ictalurus Punctatus. Fish Shellfish Immunol..

[64] Lin X., Zhou Q., Zhao C., Lin G., Xu J., Wen Z. (2019). An Ectoderm-Derived Myeloid-like Cell Population Functions as Antigen Transporters for Langerhans Cells in Zebrafish Epidermis. Dev. Cell.

[65] Rast J.P., Buckley K.M. (2013). Lamprey Immunity Is Far from Primitive. Proc. Natl. Acad. Sci. USA.

[66] Boehm T., Hirano M., Holland S.J., Das S., Schorpp M., Cooper M.D. (2018). Evolution of Alternative Adaptive Immune Systems in Vertebrates. Annu. Rev. Immunol..

[67] Mandujano-Tinoco E.A., Sultan E., Ottolenghi A., Gershoni-Yahalom O., Rosental B. (2021). Evolution of Cellular Immunity Effector Cells; Perspective on Cytotoxic and Phagocytic Cellular Lineages. Cells.

[68] Netea M.G., Schlitzer A., Placek K., Joosten L.A.B., Schultze J.L. (2019). Innate and Adaptive Immune Memory: An Evolutionary Continuum in the Host’s Response to Pathogens. Cell Host Microbe.

[69] Puhr S., Lee J., Zvezdova E., Zhou Y.J., Liu K. (2015). Dendritic Cell Development—History, Advances, and Open Questions. Semin. Immunol..

[70] Flajnik M.F. (2018). A Cold-Blooded View of Adaptive Immunity. Nat. Rev. Immunol..

[71] Bacci S., Romagnoli P. (2017). The Role of Dendritic Cells in Vertebrates: A Review. Int. Biol. Rev..

[72] Yuan S., Tao X., Huang S., Chen S., Xu A. (2014). Comparative Immune Systems in Animals. Annu. Rev. Anim. Biosci..

[73] Bao H., Liu Y., Qin J., Xu C., Hei N., Jaber J.R., Chen Q. (2011). An Immunohistochemical Study of S-100 Protein in the Intestinal Tract of Chinese Soft-Shelled Turtle, Pelodiscus Sinensis. Res. Vet. Sci..

[74] Granja A.G., Leal E., Pignatelli J., Castro R., Abós B., Kato G., Fischer U., Tafalla C. (2015). Identification of Teleost Skin CD8α^+^ Dendritic-like Cells, Representing a Potential Common Ancestor for Mammalian Cross-Presenting Dendritic Cells. J. Immunol..

[75] Alesci A., Pergolizzi S., Lo Cascio P., Fumia A., Lauriano E.R. (2021). Neuronal Regeneration: Vertebrates Comparative Overview and New Perspectives for Neurodegenerative Diseases. Acta Zool..

[76] Phrompanya P., Saenphet K., Saenphet S. (2019). Comparative Histochemical Study of the Gastrointestinal Tracts of the Nile Tilapia (*Oreochromis niloticus*) and the Hybrid Catfish (*Clarias batrachus* x *Clarias gariepinus*). Acta Histochem..

[77] Weinrauch A., Edwards S., Goss G. (2015). Anatomy of the Pacific Hagfish (*Epatatretus stoutii*). Hagfish Biology.

[78] Magid A.A. (1975). The Epithelium of the Gastro-Intestinal Tract of Polypterus Senegalus (Pisces: Brachiopterygii). J. Morphol..

[79] Verdile N., Pasquariello R., Scolari M., Scirè G., Brevini T.A.L., Gandolfi F. (2020). A Detailed Study of Rainbow Trout (*Onchorhynchus mykiss*) Intestine Revealed That Digestive and Absorptive Functions Are Not Linearly Distributed along Its Length. Animals.

[80] Yadav A.K., Srivastava P.P., Chowdhary S., Lakra W.S., Shrivastava P., Dayal R. (2014). Histological Alterations in the Intestine of Threatened Asian Catfish, Clarias Batrachus Fed with Different Types of Fats through Semi-Purified Diets. Adv. Appl. Sci. Res..

[81] Alessio A., Pergolizzi S., Gervasi T., Aragona M., Lo Cascio P., Cicero N., Lauriano E.R. (2021). Biological Effect of Astaxanthin on Alcohol-Induced Gut Damage in *Carassius auratus* Used as Experimental Model. Nat. Prod. Res..

[82] Liu G., Zhang H., Zhao C., Zhang H. (2020). Evolutionary History of the Toll-Like Receptor Gene Family across Vertebrates. Genome Biol. Evol..

[83] Rumfelt L.L., Mckinney E.C., Taylor E., Flajnik M.F. (2002). The Development of Primary and Secondary Lymphoid Tissues in the Nurse Shark *Ginglymostoma cirratum*: B-Cell Zones Precede Dendritic Cell Immigration and T-Cell Zone Formation during Ontogeny of the Spleen: Shark Lymphoid Organ Development. Scand. J. Immunol..

[84] Dong F., Song X., Xing J., Tang X., Sheng X., Chi H., Zhan W. (2021). Immunological Characteristics of Dendritic Cells Marker CD83 in Flounder (*Paralichthys olivaceus*). Fish Shellfish Immunol. Rep..

[85] Sharma B.B. Dash Toll-like Receptors (TLR) in Fish. https://www.researchgate.net/publication/324257921_TOLL-LIKE_RECEPTORS_TLR_IN_FISH.

[86] Lewis K.L., Del Cid N., Traver D. (2014). Perspectives on Antigen Presenting Cells in Zebrafish. Dev. Comp. Immunol..

